# Application of New Polymer Soil Amendment in Ecological Restoration of High-Steep Rocky Slope in Seasonally Frozen Soil Areas

**DOI:** 10.3390/polym16131821

**Published:** 2024-06-27

**Authors:** Zengkang Lu, Chenglong Yu, Huanan Liu, Jiquan Zhang, Yichen Zhang, Jie Wang, Yancheng Chen

**Affiliations:** 1 College of Jilin Emergency Management, Changchun Institute of Technology, Changchun 130012, China; 20221311204@stu.ccit.edu.cn (Z.L.); zhangyc@ccit.edu.cn (Y.Z.); 20221311210@stu.ccit.edu.cn (J.W.); 2 School of Prospecting and Surveying, Changchun Institute of Technology, Changchun 130021, China; liuhuanan@ccit.edu.cn (H.L.); 2101461109@stu.ccit.edu.cn (Y.C.); 3 School of Environment, Northeast Normal University, Changchun 130024, China; zhangjq022@nenu.edu.cn

**Keywords:** seasonally frozen soil area, rocky slope, polyacrylamide, carboxymethyl cellulose, vegetation growth

## Abstract

In seasonally frozen soil areas, high-steep rocky slopes resulting from open-pit mining and slope cutting during road construction undergo slow natural restoration, making ecological restoration generally challenging. In order to improve the problems of external soil attachment and long-term vegetation growth in the ecological restoration of high-steep rocky slopes in seasonally frozen areas, this study conducted a series of experiments through the combined application of polyacrylamide (PAM) and carboxymethyl cellulose (CMC) to assess the effects of soil amendments on soil shear strength, water stability, freeze–thaw resistance, erosion resistance, and vegetation growth. This study showed that the addition of PAM-CMC significantly increased the shear resistance and cohesion of the soil, as well as improving the water stability, freeze–thaw resistance, and erosion resistance, but the internal friction angle of the soil was not significantly increased after reaching a certain content. Moderate amounts of PAM-CMC can extend the survival of vegetation, but overuse may cause soil hardening and inhibit vegetation growth by limiting air permeability. It was observed by a scanning electron microscope (SEM) that the gel membrane formed by PAM-CMC helped to “bridge” and bind the soil particles. After discussion and analysis, the optimum application rate of PAM-CMC was 3%, which not only improved the soil structure but also ensured the growth of vegetation in the later stage under the optimum application rate. Field application studies have shown that 3% PAM-CMC-amended soil stably attaches to high-steep rocky slopes, with stable vegetation growth, and continues to grow after five months of freeze–thaw action, with no need for manual maintenance after one year.

## 1. Introduction

With the increasing awareness of human environmental protection, people’s demand for environmental quality is increasingly high, the contradiction between engineering construction and environmental protection is becoming obvious, and the traditional slope protection technology of rocky slope engineering (such as spray concrete [1,2], hanging net slurry spraying [3], geocell reinforcement [4], etc.) has a poor greening effect and ecological environmental benefits and cannot well meet the requirements of environmental protection. In seasonally frozen areas, the ecological restoration of high-steep rocky slopes is a task of great significance, due to the seasonally frozen areas having large temperature changes and frequent alternation between the freezing and thawing of the soil, resulting in high-steep rocky slopes susceptible to landslides, collapse, and other disasters [5,6,7]. Therefore, the problems of enhancing the attachment capacity of the external soil and the long-term survival of vegetation have become important research directions for the ecological restoration of high-steep rocky slopes at present.

Soil amendments commonly used in the ecological restoration of high-steep rocky slopes include cement, fly ash, lime, organic cementation materials, and polymers. Cement can increase the strength and stability of the soil, but it is used in large quantities and has a high cost [8]. Fly ash is often used as a soil amendment in agriculture, where it has good adsorption properties and can improve soil structure and fertility [9]. Lime improves the mechanical properties of the soil, reducing its hydrophilicity and increasing its cohesive strength [10,11]. Polymers are unique among these soil amendments in that they are renewable and readily available and also improve soil mechanics [9], water retention, and water stability [12,13], as well as facilitating plant germination and root extension [14,15,16]. Currently, in the ecological restoration of high-steep rocky slopes, the research on polymers has mainly focused on their effects on the freeze–thaw cycle performance of the soil, the effect of cooperation with other soil improvement materials, and the promotion of plant growth.

Previous studies have shown that polyacrylamide (PAM) is a soil amendment with an excellent water and moisture retention capacity [17,18,19]. It contains a long molecular chain with abundant hydrophilic groups—amide groups (-CONH_2_)—which can interact with water molecules through hydrogen bonding, giving PAM a strong water absorption capacity [20]. It can increase the soil’s bonding and cohesion, improve the soil’s resistance to freezing and scouring, and thus increase the resistance to sliding and scouring [21,22,23,24] of high-steep rocky slopes. In addition, polyacrylamide can keep the soil moist and promote the growth of plants, which helps the recovery of slope vegetation and the improvement of the ecological environment. Carboxymethyl cellulose (CMC) is a biodegradable soil conditioner with excellent consolidation and cohesion properties [25,26]. It can eventually decompose into carbon dioxide and water through the action of cellulase and microbial utilization. Its degradation rate varies with temperature and soil properties. Additionally, it is a hydrophilic polymer. Its hydrophilic group, carboxymethyl (-CH2COOH), imparts good water solubility and strong water absorption due to its negative charge [27]. In the ecological restoration of slopes, carboxymethyl cellulose can improve soil aggregation, cohesion, and erosion resistance [28]; increase soil stability and slip resistance [29]; reduce soil erosion; and promote the growth of vegetation and the development of the root system, which is conducive to the restoration of slopes and improvement of the ecological environment. Therefore, it is important to study the application of polyacrylamide and carboxymethyl cellulose in the ecological restoration of high-steep rocky slopes in seasonally frozen soil areas in order to improve the stability of slopes and restore the ecological environment.

Currently, PAM and CMC have been studied in the context of rocky and sandy slopes. Xu et al. [9] conducted ecological restoration on rocky slopes in cold, high-altitude areas using soil substrates like PAM and CMC. They found that moderate amounts of PAM and CMC could enhance soil aggregates and the field capacity. Yuan et al. [28,30] investigated the effects of PAM and CMC on the erosion resistance of sandy slopes and examined the adsorption kinetics of these amendments on sand grains through rainfall erosion tests. Their results demonstrated that incorporating these amendments significantly reduced water infiltration rates and average sand production in sandy slopes, with amendments being adsorbed onto the sand grains via chemical complexation. Yang et al. [31] conducted a series of laboratory tests and field studies on silty sand slopes using PAM and CMC. The results demonstrated that the cohesion, water susceptibility, and erosion resistance of silty sand were significantly improved, and the soil loss was greatly reduced as the concentration of the amendments increased. Zhao et al. [32] investigated the effect of PAM and CMC on the shear strength of sandy loess and analyzed the amendment mechanism from a microscopic perspective. Their results indicated that the addition of these amendments facilitated the combination and adsorption of sand particles in the soil, forming larger aggregates and thereby improving the shear strength of the sandy loess.

Seasonal frozen soil areas experience large temperature fluctuations due to the alternation between freezing and thawing. The properties of a polymer can change under multiple freeze–thaw cycles, thereby affecting its durability. This study investigated the effect of a PAM-CMC amendment on powdery silty clay under freeze–thaw conditions specific to the study area’s environment and evaluated its freeze–thaw resistance through quantitative analyses. In addition, building on previous research, the effects of the combined application of PAM and CMC on the soil and vegetation growth were investigated through a series of laboratory tests (direct shear tests, water stability tests, erosion resistance tests, etc.); the properties of improved soil, such as shear strength, cohesion, internal friction angle, water stability, erosion resistance, etc., were evaluated; and the microstructure of the improved soil was observed using a scanning electron microscope (SEM). The stability mechanism was analyzed, and finally, the effectiveness of PAM-CMC in the ecological restoration of high-steep rocky slopes in seasonally frozen soil areas was verified by field application.

## 2. Materials and Methods

### 2.1. Materials

#### 2.1.1. Soil Sample Collection

**Figure 1:**
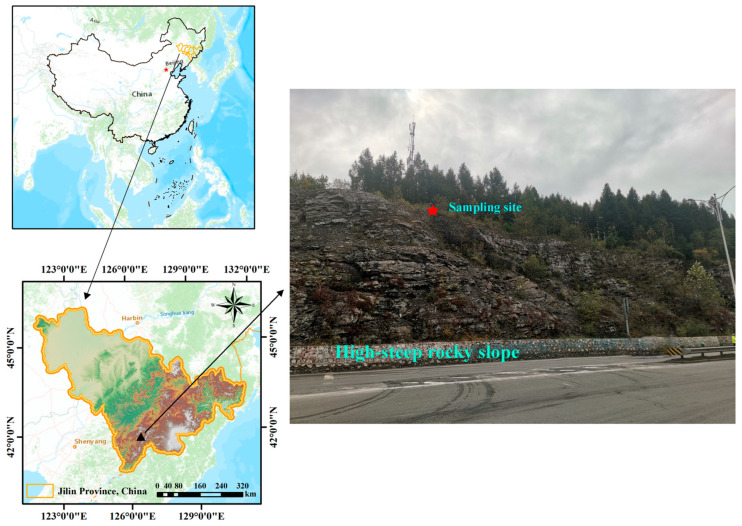
Soil sample collection site.

**Figure 2:**
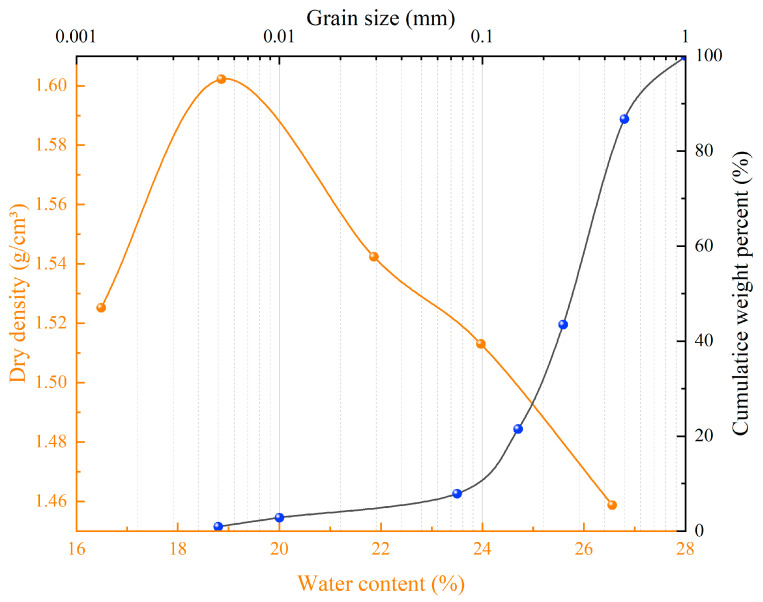
Compaction curves and particle size distribution.

In this study, the soil used for testing was obtained from a natural hillside surrounding a high-steep rocky slope in the urban area of Baishan City, Jilin Province, China (Figure 1), which was also used for field validation in this study. Its geographical coordinates were 126°28′18″ E, 41°57′51″ N. The formation of the high-steep rocky slope was due to the early construction of the highway cutting the slope and destroying the original mountain, with a slope angle of about 50–65°, and the rocky slope was exposed, with extremely sparse vegetation, and there were only a few vegetation growths in a small number of platforms or at the residual soils in the rock crevices. The overall stability of the slope was good, but there were some pumice and dangerous rocks on the slope surface. The obtained soil samples (including fine rocks and roots) were first air-dried, crushed, and passed through a 2 mm sieve and then tested for their main properties according to GB/T 50123-2019 [33] (the Chinese standard for geotechnical testing methods), and the basic properties are shown in Table 1. The results of the compaction test and grain size distribution are shown in Figure 2, which shows that most of these soil particles were between 0.075 and 0.5 mm and were classified as silty clay according to the Unified Soil Classification System.

#### 2.1.2. Preparation of PAM-CMC

**Figure 3:**
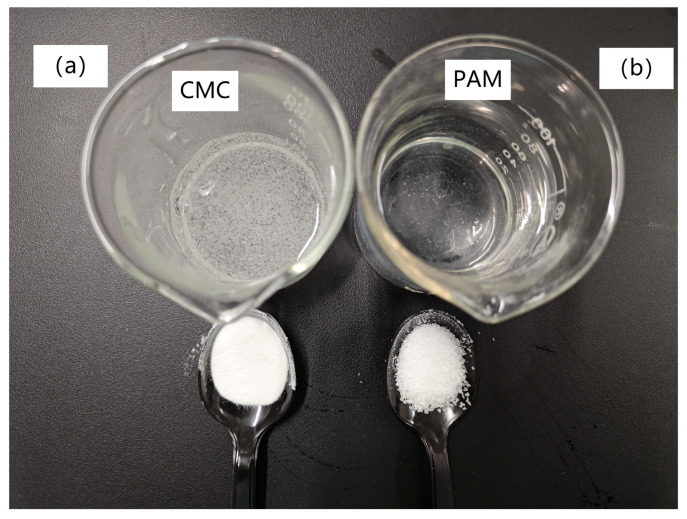
Photographs of CMC and PAM powders and solutions.

Polyacrylamide (PAM) is a water-soluble polymer that appears as a white granular powder [34], widely employed to enhance the stability and erosion resistance of clay and sandy soils. Carboxymethyl cellulose (CMC) is a water-soluble polymer gel comprising numerous hydroxyl and carboxyl groups, renowned for its outstanding biocompatibility [35] and biodegradability. CMC exhibits excellent water absorption and adhesion properties, can be cured at room temperature (20 °C), and finds extensive applications as a water-retaining agent and binder for agricultural soil enhancement. In this study, CMC (Figure 3a) and PAM (Figure 3b) were mixed and applied in soil properties and plant growth studies, and their properties are shown in Table 2. Viscosity was determined by a digital six-speed rotational viscometer (MK-6ST) for 350 mL of a 1% solution of both CMC and PAM. Since both CMC and PAM are highly hygroscopic materials, this study calculated the hygroscopic water content (W_h_) based on the method in the literature [36,37] and conducted hygroscopicity tests in indoor environments to precisely determine the dosage of CMC and PAM.

A PAM-CMC amendment is a mixed solution made by diluting PAM and CMC separately and then mixing them. Previous studies have shown that excess PAM-CMC covers most soil particles and aggregates, fills voids [9], and can harden [31] the soil and inhibit vegetation growth. A pre-experiment was carried out to improve the soil using the PAM-CMC formulation in the present study, and it was found that when PAM-CMC was applied at up to 5%, hardening of the soil took place at 12 h of curing, and the soil was severely caked, which was not favorable for the growth of the vegetation, so PAM-CMC contents of 1%, 2%, 3%, and 4% were used to carry out the experimental study.

The preparation method was as follows: (i) CMC and PAM powders were dissolved in water and diluted to a concentration of 1 g/100 mL, respectively, and stirred using a mixer at 200 r/min to ensure that they were completely dissolved to obtain a 1% solution of CMC and PAM, respectively; (ii) in order to avoid the entanglement of molecules in the mixed solution, quantitative amounts of the CMC and PAM solutions were diluted separately using 100 mL of pure water as a solvent, and finally, a further diluted PAM solution and CMC solution were added slowly while 200 mL of pure water was stirred in the mixer. In this study, the total amount of PAM-CMC solution prepared each time was 400 mL, which corresponded to the addition of 4–16 mL of the initially diluted PAM and CMC solutions (the content of both the added PAM and CMC solutions was half) per 400 mL of the aqueous solution, so the solutions reached concentrations of PAM-CMC of 1%, 2%, 3%, and 4% (the two solutions prepared in (i) were solutes). The amendment was a colorless, odorless, non-toxic gel-like solution with a viscosity of 12.7 mPa-s at 4% and a pH of 7.2.

### 2.2. Laboratory Tests

#### 2.2.1. Direct Shear Test

**Figure 4:**
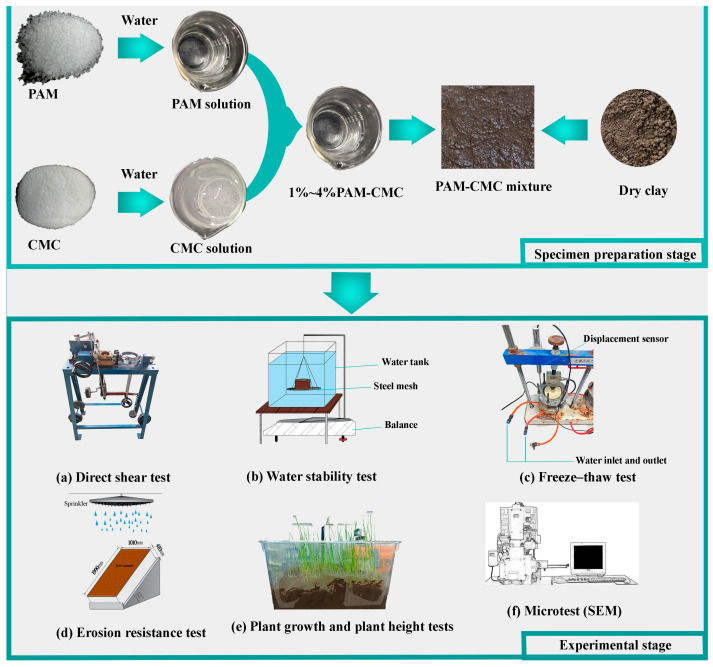
Schematic diagram of the laboratory test project.

The device used for the tests was an electrically operated equal-strain direct shear device (Figure 4a), and the effect of the PAM-CMC content (0%, 1%, 2%, 3%, and 4%) on the mechanical behavior of the amended soil was investigated through a series of fast shear tests. For these tests, the direct shear test specimens were prepared with the optimum water content and maximum dry density, with each specimen measuring 20 mm in height and 6.2 mm in diameter. Sixty specimens were prepared for various PAM-CMC contents and curing times and subsequently underwent rapid shear testing under vertical stresses of 100, 200, 300, and 400 kPa after 48 h of curing. The shear rate was set at 0.8 mm/min.

#### 2.2.2. Water Stability Test

While the buoy method, as outlined in GB/T 50123-2019, is typically employed to assess soil water stability, the weight measuring method [15,38] was adopted for this test due to the difficulty in obtaining precise measurements through the former approach. The apparatus comprised three components: a balance, a steel mesh (with apertures of 1 cm^2^), and a water tank (Figure 4b). All specimens were compacted to the optimum water content and the maximum dry density of the soil, with a diameter of 61.8 mm and a height of 40 mm. The contents of PAM-CMC in the specimens were 0%, 1%, 2%, 3%, and 4%, and the curing time of the specimens was tested on the 3rd and 7th day of curing naturally in the room. Balance readings were taken at 10 min intervals during the tests and the disintegration pattern of the soil was recorded, and the tests were finished when the specimens were completely disintegrated or when the amount of disintegration exceeded 60%. In this study, the disintegration ratio and disintegration rate in water were used as evaluation indexes of the water stability of the soil [39] with the following equations, respectively:

(1) $$R_{d}\left(t\right)=\frac{m_{0}-m_{t}}{m_{0}}\times 100\%$$

(2) $$V\left(t\right)=\frac{R_{d}\left(t\right)-R_{d}\left(t^{\prime }\right)}{t-t^{\prime }}$$

where $R_{d}\left(t\right)$ is the disintegration ratio of the soil sample at *t* min; *m*_0_ is the balance reading of the soil sample immersed in water for 0 min (g); *m_t_* is the balance reading of the soil sample in water for *t* min (g); and *V*(*t*) is the disintegration rate at *t* − *t*’ min (%/min).

#### 2.2.3. Freeze–Thaw Test

The freeze–thaw device used in this study was a refrigerator, and a freeze–thaw displacement measurement device, as shown in Figure 4c, was used to measure the frost heave amount and thaw settlement of the specimens during the frost heave and thaw-settlement. Soil samples were compacted to the maximum dry density, frozen at −20 °C for 12 h, and then thawed at 20 °C for 12 h. The test was divided into two major processes of frost heave and thaw settlement, and the evaluation indexes were characterized by the frost heave ratio and the thaw settlement coefficient, respectively. According to the average temperature and freeze–thaw conditions from October to March every year in Changchun City, this study undertook six freeze–thaw cycle tests, measured the amount of frost heave and thaw settlement in the six freeze–thaw cycles, and carried out three replicated tests to obtain the mean and standard deviation of the frost heave ratio and thaw settlement coefficient in order to evaluate the freeze–thaw resistance properties of PAM-CMC in the soil. The frost heave ratio and the thaw settlement coefficient of the soil samples were calculated as shown in Equations (3) and (4):

(3) $$\eta =\frac{\Delta h}{H_{f}}\times 100\%$$

(4) $$\alpha_{0}=\frac{\Delta h_{0}}{h_{0}}\times 100\%$$

where *η* is the frost heave ratio (%); Δ*h* is the total frost heave amount during a single freeze (mm); and $H_{f}$ is the freezing depth (mm), i.e., the initial height of the specimen before freezing, excluding the frost heave amount. $\alpha_{0}$ is the thaw settlement coefficient (%); $\Delta h_{0}$ is the total settlement during a single thaw (mm); and $h_{0}$ is the height of the specimen before thawing and melting, i.e., the height of the specimen after being frozen and stabilized, including the frost heave amount (mm).

#### 2.2.4. Erosion Resistance Test

The simulated rainfall test setup is shown in Figure 4d, which could be adjusted for rainfall intensity, and the simulated rainfall tests were conducted in PAM-CMC-amended soils with specimens of 0%, 1%, 2%, 3%, and 4% and specimen dimensions of 200 mm in length, 100 mm in width, and 40 mm in height. All specimens were compacted to the optimum water content and maximum dry density of the soil and then cured in a moist environment for 48 h. All specimens were fixed at a 53° (1:0.75) inclination, and the rainfall intensity was adjusted to 0.9 L/min to simulate the heavy rainfall conditions of the study area for a duration of 30 min. Upon completion of the experiment, the soil remaining on the slope was separated from the washed-away soil and dried in an oven, and then the erosion rate [15] was calculated using the following equation:

(5) $$R=\left(1-\frac{m_{r}}{m_{0}}\right)\times 100\%$$

where *R* is the erosion rate (%); $m_{r}$ is the dry weight of the remaining soil (g); and $m_{0}$ is the initial dry weight of the specimen (g).

#### 2.2.5. Plant Growth and Height Test

Plant growth tests and plant height measurements were performed on five groups of samples of 0%, 1%, 2%, 3%, and 4% PAM-CMC (Figure 4e). Plant height was the main indicator of plant growth and was measured from the 7th day of the experiment. The height of the plant was recorded by measuring the distance from the soil surface to the top of the plant’s leaves, and values were taken using the mean and standard deviation. Ryegrass (*Lolium perenne* L.) was used in this study in 20 cm × 20 cm × 15 cm planting boxes, and due to the fast growth rate of the plants, an equal amount of water was replenished to each planting box every 3 days in this experiment based on the moisture levels of the soil.

#### 2.2.6. SEM Test

The test setup is shown in Figure 4f, and the specimens taken for the scanning electron microscope (SEM) test measured 1 cm × 1 cm × 2 cm. The microstructure of the soil samples was observed in this test using specimens treated with PAM-CMC solutions at concentrations of 0% and 4%, respectively. For the made specimen, we first used liquid nitrogen to quickly freeze the soil sample so that the moisture in the soil became non-crystalline ice without volume expansion, and then we used a vacuum freeze dryer to sublimate the non-crystalline ice in the soil sample so as to achieve the purpose of drying the specimen without destroying its structure. The specimen was then fractured to form a new fracture surface of 1 cm^2^ to a height of about 1 cm. The micro-morphology of the specimen was observed on the fracture surface at magnifications of 800, 2000, and 5000 times using scanning electron microscopy (SEM).

## 3. Results

### 3.1. Results of Direct Shear Tests

**Figure 5:**
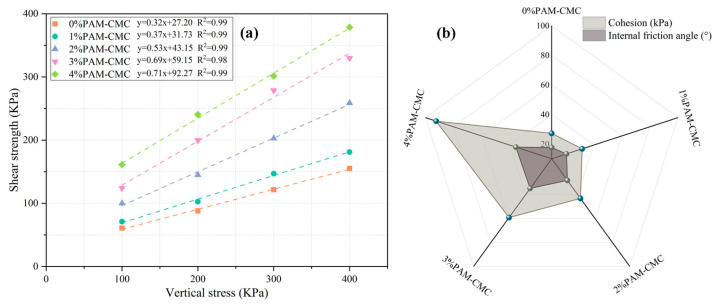
Shear strength (**a**), cohesion, and internal friction angle (**b**) for different PAM-CMC contents.

The results of the direct shear tests are shown in Figure 5a. The results show that the addition of PAM-CMC significantly increased the shear strength of the soil, and the increase in the shear strength of the specimens increased with the increase in vertical stress. It can be seen that at 0% and 1%, the shear strength slightly increased, and at a 100 kPa vertical stress, the shear strength of the PAM-CMC content of ≥2% increased by at least 64.57% compared with 0%, and the magnitude of the increased shear strength by PAM-CMC incorporation was even more pronounced at higher vertical stresses. The variation in the cohesion and internal friction angle with the PAM-CMC content is shown in Figure 5b, where it can be seen that the cohesion increased almost exponentially with an increasing PAM-CMC content, while the internal friction angle also exponentially increased at application rates of ≤3%, and the internal friction angle at a treatment rate of 4% was not much different compared with 3%. According to the Moore–Cullen criterion, it can be seen that the increase in shear strength was mainly due to the increase in inter-particle cohesion and the internal friction angle, while the shear strength of the specimens at high concentrations of PAM-CMC was less affected by the internal friction angle.

### 3.2. Results of Water Stability Tests

#### 3.2.1. Disintegration Ratio and Disintegration Rate

**Figure 6:**
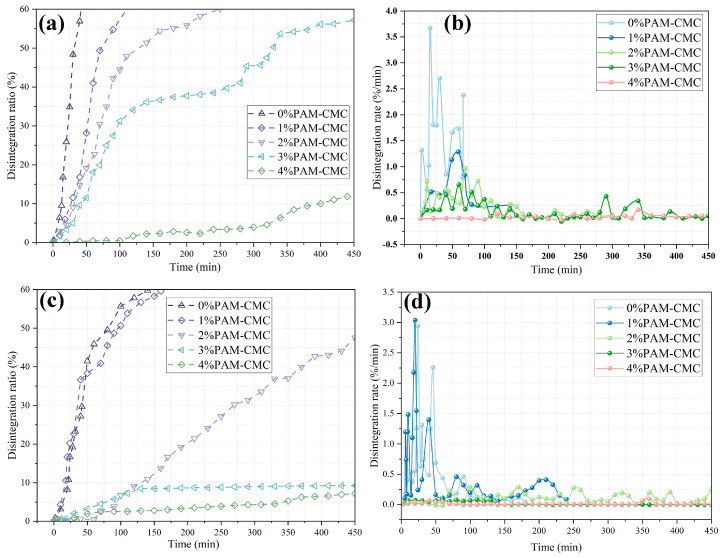
Variation in disintegration ratio and disintegration rate of the specimens with time: disintegration ratio (**a**) and disintegration rate (**b**) for 3 days of curing; disintegration ratio (**c**) and disintegration rate (**d**) for 7 days of curing.

For the specimens cured for 3 days, it can be seen in Figure 6a that the disintegration ratios of 0–3% PAM-CMC all continuously increased and exceeded 60% with time, while that of 4% PAM-CMC remained below 20% at longer disintegration times, which suggests that disintegration continued to increase at lower concentrations of PAM-CMC after relatively short curing times, while high concentrations of PAM-CMC made the specimens gel quickly and improved the stability of the specimens in water. As can be seen in Figure 6b, the addition of PAM-CMC significantly improved the water stability of the soil, with a maximum disintegration rate of 3.67%/min for 0% PAM-CMC, compared with the disintegration rate of 0.1~1.5%/min for the 1–3% PAM-CMC specimens, and a stable rate of 0.25%/min or less for the 4% PAM-CMC specimens. As can be seen in Figure 6c, the end of disintegration of the specimens cured for 7 days was prolonged, and the low-concentration (≤1%) PAM-CMC specimens continuously disintegrated until the end of the test; the 2% PAM-CMC specimens still behaved with continuous disintegration, but the amount of disintegration was relatively small; and the 3–4% PAM-CMC specimens reached a stable condition after a slow disintegration in the early stage, and the final disintegration ratio was below 10%. As can be seen in Figure 6d, the low-concentration (≤1%) PAM-CMC specimens underwent very unstable disintegration in the early stage, and the higher-concentration specimens disintegrated more slowly and at a lower disintegration rate. The maximum disintegration rate for both the 0% and 1% PAM-CMC specimens exceeded 2%/min, while those of specimens with ≥2% PAM-CMC were below 0.5%/min. This further indicates that the soil with added PAM-CMC exhibited good water stability.

#### 3.2.2. Characteristics of Disintegration Pattern

In this test, the change in disintegration pattern was recorded to evaluate the water stability of the specimens by immersing them in hydrostatic water at different moments of immersion, and the specimens with 0% and 4% PAM-CMC were studied to compare their disintegration patterns.

**Figure 7:**
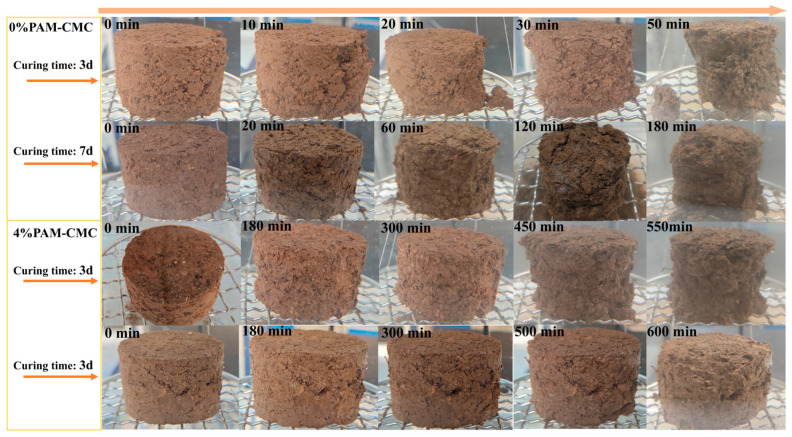
Images of unamended soil and amended soil with 4% PAM-CMC.

As can be seen in Figure 7, a large crack appeared on the surface of the 0% PAM-CMC specimen cured for 3 days after 10 min of immersion in water, and the soil in the upper part of the crack was dislodged due to loosening. At 20 min, the cracks continued to develop and eventually all peeled off, and a portion of the soil separated from the bottom of the specimen, and the rest of the soil sample was gradually stripped off due to the same crack development. However, the 4% PAM-CMC specimens cured for 3 days did not show any significant cracks, and the small soil clods on the outside of the specimens were slowly detached due to water absorption and swelling as the soaking time increased.

For the 0% PAM-CMC specimens cured for 7 days, some small cracks appeared after 20 min of immersion, in which the clods were detached with time, and several larger cracks appeared in the specimens at 120 min, and eventually all the clods outside the cracks were detached. In contrast, the 4% PAM-CMC specimens cured for 7 days began to swell at 300 min, and the loose soil on the surface began to fall off after 500 min, without significant disintegration.

### 3.3. Results of Freeze–Thaw Test

**Figure 8:**
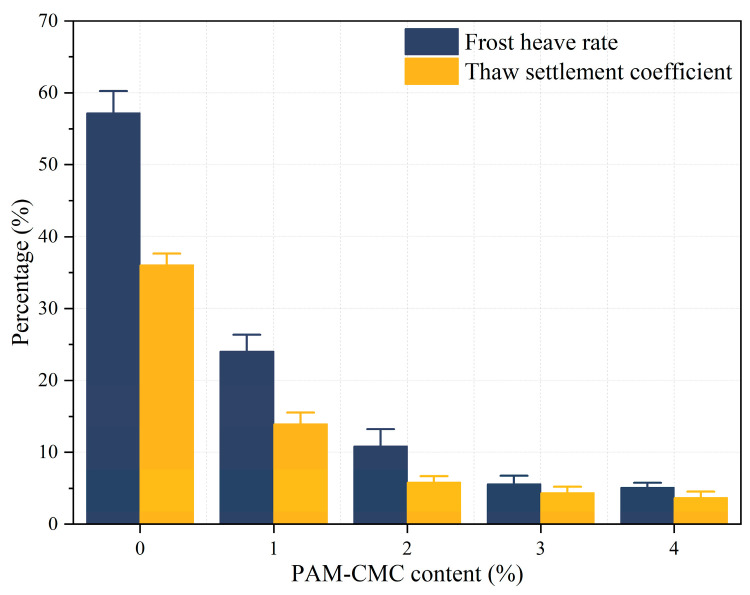
Mean frost heave rate and mean thaw settlement coefficient for different PAM-CMC contents.

The frost heave and thaw settlement displacements of the soil samples were measured after six freeze–thaw cycles, and the results are shown in Figure 8. The frost heave rate and thaw settlement coefficients of the unimproved specimen (0% PAM-CMC) were significantly higher than those of the improved soil samples, which were 57.2% and 36.06%, respectively. Followed by 1% PAM-CMC, the frost heave rate and thaw settlement coefficient were 24.02% and 13.97%, which were 58% and 61.26% lower than those of the unimproved specimens, respectively, while the frost heave rate and thaw settlement coefficient of 2–4% were lower (≤15%), which indicated that the anti-freezing and thaw-thawing performance was significantly improved by the addition of a certain amount of PAM-CMC.

### 3.4. Results of Erosion Resistance Tests

#### 3.4.1. Characteristics of Surface Erosion

**Figure 9:**
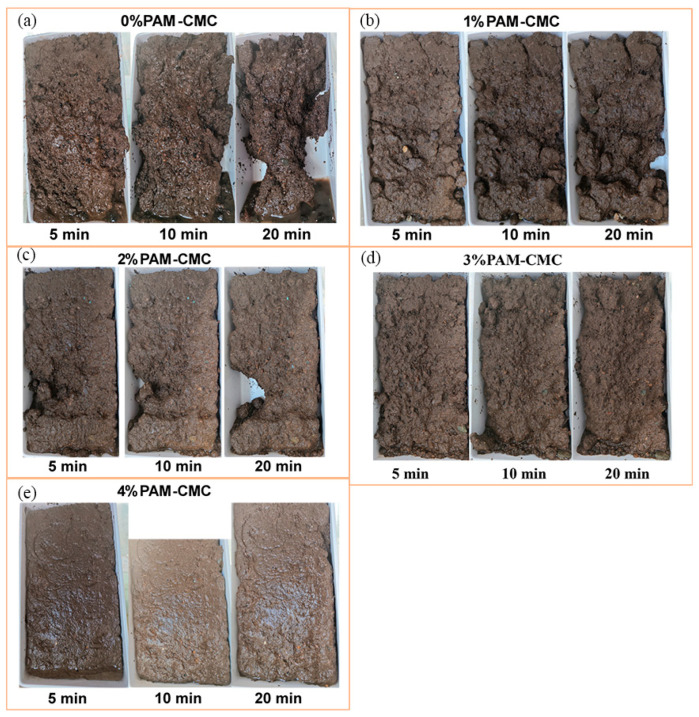
Images of erosion process of specimens with different PAM-CMC contents.

The surface erosion process of the specimens is shown in Figure 9. In general, the soil with the addition of PAM-CMC had better stability and erosion resistance for the 0% PAM-CMC specimens, and the erosion resistance of the specimens increased with the increase in the PAM-CMC content. In the 0% PAM-CMC specimen under rainfall, the surface was gradually washed away, the permeability speed was accelerated and then eroded by the rainfall, the pore structure was destroyed so that precipitation could penetrate into the soil interior more quickly, the soil surface and the interior were washed away, and finally, complete disintegration occurred due to the instability of the soil structure (Figure 9a). The soil with 1% PAM-CMC added was different in that the surface was washed away with soil particles, while the agglomerates, whose erosion resistance [30] was improved by the binding effect of PAM-CMC, had their surfaces continually washed away by rainfall, and in the end, only the large agglomerates were not washed away (Figure 9b). Under continuous rainfall, erosion pits appeared in the 2% PAM-CMC soil samples, and eventually, the rainwater eroded to the bottom of the soil samples, which made the soil unstable and slumped (Figure 9c). While the 3% PAM-CMC surface was washed away, it still did not erode into the interior of the soil, and many small pits appeared on the surface (Figure 9d). The soil with 4% PAM-CMC added was more stable, and the surface was harder due to the maintenance for 48 h. After 20 min, only some shallow erosion pits appeared on the surface, and a small portion of the soil was eroded at the edge. After 30 min, only a small amount of the soil was washed away, no cracks appeared, and the soil’s structure was still relatively stable (Figure 9e).

#### 3.4.2. Erosion Rate

**Figure 10:**
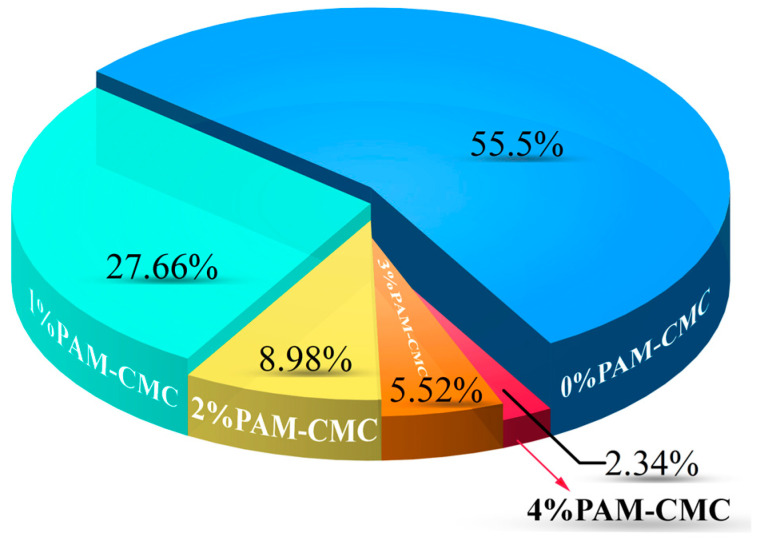
Percentages of erosion rates for specimens with different PAM-CMC contents.

Figure 10 illustrates the erosion rate percentages of the specimens with varying PAM-CMC contents subjected to rainfall erosion, revealing that the erosion rate decreased approximately exponentially as the PAM-CMC content increased. Rainfall caused the surfaces of the specimens to erode and then erode further into the interior, and when runoff loosened the soil, it was washed away, further enlarging the erosion pits and gullies. The erosion rate of the 0% PAM-CMC specimens was 34.33%, representing 55.5% of the total erosion rate. Moreover, when the PAM-CMC content was ≥2%, the erosion rate decreased by at least 83.83% compared with the unamended soil, which had far less erosion than the unamended soil, indicating significantly reduced erosivity.

### 3.5. Results of Plant Growth and Plant Height Test

**Figure 11:**
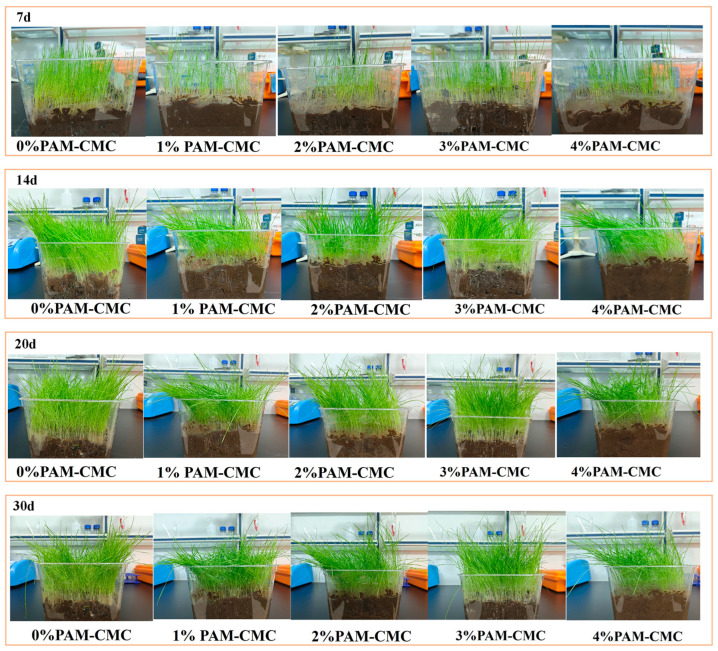
Plant growth of specimens with different PAM-CMC contents.

**Figure 12:**
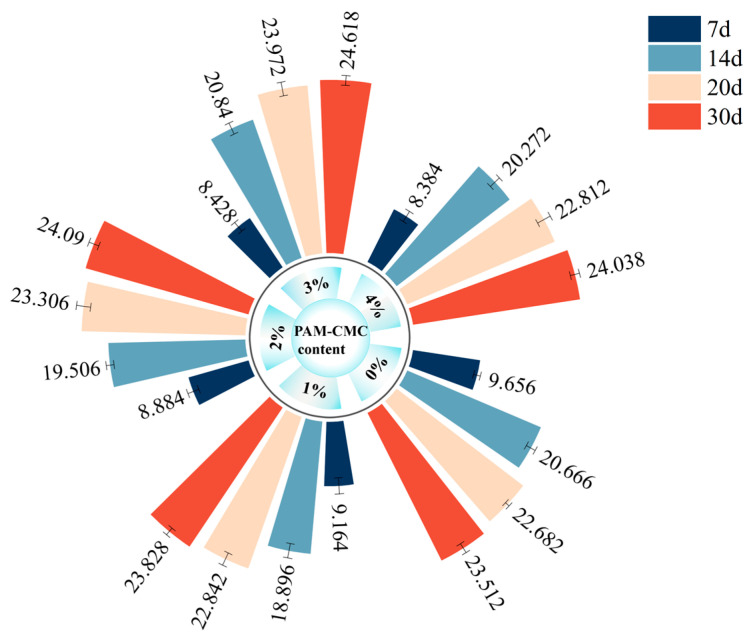
Measurements of average height of plant growth (cm).

Figure 11 shows the plant growth in the amended soil with different PAM-CMC contents. The results show that the early seed germination in 0% PAM-CMC was fast, and the average plant height was large, while the opposite was true for the seed germination rate in the amended soil, and the average plant height decreased with an increasing PAM-CMC content. At the early stage of plant growth, 0% PAM-CMC germinated the fastest, and all plants grew to similar heights, while plants in the amended soil germinated more slowly, with some seeds still starting to germinate at 7 days of growth, and some plant heights were significantly higher than the average. As can be seen from the average height of plant growth in Figure 12, the average plant height in the amended soil increased with time, and at the time of plant growth up to 14 days, the maximum average plant height in the amended soil was 3% PAM-CMC, followed by 4% PAM-CMC, and, lastly, 2% PAM-CMC and 1% PAM-CMC. The plants were essentially stable by 20 days of growth, and by 30 days of growth, the 0% PAM-CMC and 1% PAM-CMC plants began to wilt, with some leaves yellowing and leaning and were significantly better in 1% PAM-CMC than in 0% PAM-CMC. However, 2–4% of the plants continued to thrive, indicating that the addition of PAM-CMC not only increased plant height but also prolonged plant longevity.

### 3.6. Microscopic Characteristics of Amended Soil

**Figure 13:**
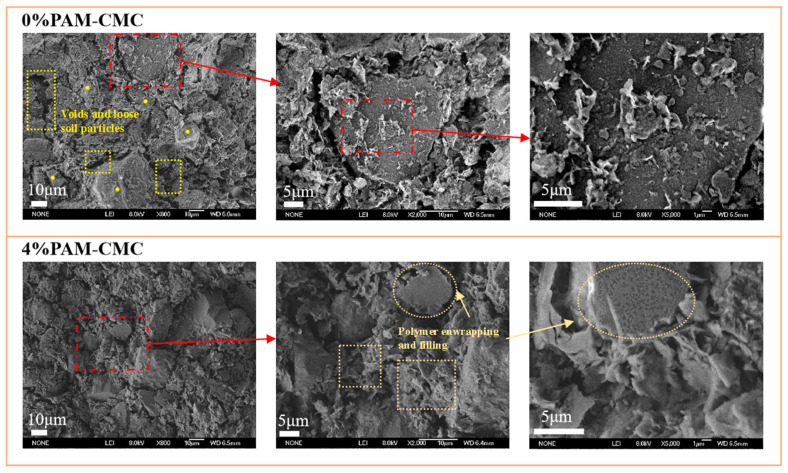
SEM images of unamended soil and 4% PAM-CMC-amended soil.

The microstructures of the 0% and 4% PAM-CMC-treated specimens are shown in Figure 13. It can be clearly seen that the unamended soil was porous with well-defined particle boundaries and loose soil particles. When the soil was treated with 4% PAM-CMC, most of the pores were filled with a gel-like substance, which blurred the boundaries of the modified soil particles, and the small gaps between the soil particles were also filled with PAM-CMC, and the soil particles were enwrapped in the polymer, which created a cementation layer and a “bridge” on the surfaces of the soil particles.

## 4. Discussion

### 4.1. Effect of PAM-CMC on Shear Resistance of Soil

Polymeric soil amendments improve soil through a variety of mechanisms including forming gelling substances [13,40], increasing soil cohesion [12,41,42], and preventing the migration of soil particles [14]. PAM-CMC forms an adsorption force [43] with soil particles through the group bond in the molecular structure, thus gelling the soil particles and forming the agglomerate structure, and the hydrophilic groups (-OH and -COONa) in it, because of the hydrogen bonding and van der Waals force [31], make PAM-CMC interlock to form a network structure; thus, it can be combined with a large amount of water to form a hydrogel, which can be adsorbed and bonded with the surfaces of soil particles to increase the connecting force between soil particles to form a more stable soil structure [44]. These gelling substances can adsorb and combine with the surfaces of soil particles, increasing the connectivity between soil particles and forming a more stable soil structure [45,46]. In addition, PAM-CMC prevented the migration of soil particles, as evidenced by their formation of a cemented layer and filling of the soil pore structure, an effect that became more pronounced when the concentration of PAM-CMC was high, and they made the agglomerates link with each other to become a single unit, which increased the cohesion and internal friction angle of the soil (Figure 5b). In the SEM images (Figure 13), it can be seen that the soil surface was enwrapped with PAM-CMC, and the surrounding soil was also “bridged” together, which enhanced the bonding of PAM-CMC with the soil, and this gel–soil bonding improved the shear strength of the soil. When the PAM-CMC content was increased from 3% to 4%, the cohesion of the soil increased from 59.15 KPa to 92.27 KPa, and the internal friction angle increased from 34.83° to 35.48°. Therefore, the shear strength of the soil was still improved at high levels of PAM-CMC, so the optimum application rate of PAM-CMC was 4%.

### 4.2. Effect of PAM-CMC on Water Stability of Soil

The incorporation of PAM-CMC and a sufficient curing time resulted in the improved stability of the soil in water, and the structural integrity of the soil itself was particularly important when the soil was under immersion [47,48]. Unamended soils have higher permeability and poorer cohesion and eventually disintegrate altogether due to water immersion, whereas soils with added PAM-CMC maintain their structural integrity due to inter-particle cementation and have lower permeability [17] after the pores have been filled, and water flows into the interior of the soil at a slower speed, which disintegrates slowly as the immersion time increases, and the small external clods disintegrate only after they have been disintegrated. When the curing time was short, the cohesive aggregation of the soil was low [49], whereas the addition of PAM-CMC led to an increase in the cohesion of the soil (Figure 5b), which resulted in an increase in the disintegration resistance of the soil. The water stability of the soil was only significantly improved at a sufficient curing time (>3 days) with a PAM-CMC content of ≥3% (Figure 6c), which was due to the fact that soil with a lower PAM-CMC content still undergoes successive disintegration when subjected to immersion. Therefore, in this study, the optimum application rate of PAM-CMC was 3% in order to ensure that the soil had a high degree of water stability and, at the same time, that it had a certain curing time.

### 4.3. Effect of PAM-CMC on the Freeze–Thaw Resistance of Soil

During freezing–thawing, PAM-CMC may reduce soil loosening [50] by improving the structure of the soil and reducing the gaps between soil particles to reduce water permeation. PAM-CMC has good water retention; it adsorbs and retains water in the soil, and water retention reduces the freezing of soil particles, which reduces the freezing pressure and frost heave damage [51,52] between soil particles. In addition, the gelling substance formed by PAM-CMC fills the voids between the soil particles, reduces the permeability of the soil, and enhances the cohesion and bonding between the soil particles to form a stable soil structure, which may also help to reduce the movement of water in the soil, slow down the migration of water and the settling of the soil in the freezing and thawing process, and improve the freeze–thaw resistance of the soil. In this study, the frost heave rate of the soil decreased from 10.83% to 5.6%, and the thaw settlement coefficient decreased from 5.84% to 4.87% when the PAM-CMC content was varied from 2% to 3%, whereas the frost heave rate of the soil decreased by only 0.47% and the thaw settlement coefficient decreased by only 0.68% when the PAM-CMC content was varied from 3% to 4%. Therefore, 3% PAM-CMC was the optimum application rate for the freeze–thaw resistance of the soil.

### 4.4. Effect of PAM-CMC on Erosion Resistance of Soil

During erosion, PAM-CMC may reduce the permeability of the soil surface by forming a protective or gel layer over the soil surface, reducing the direct impact of rainwater on the soil, which can reduce the amount of runoff from the soil surface and reduce the degree of soil erosion [30,31]. Unamended soils are loosely structured due to the relatively large number and size of pores between particles, which are easily eroded to the interior [15] under the impact of rainfall, and the soil on the surface moves downward with the runoff due to raindrops. The phenomenon of rainfall impacts on amended soils is generally manifested as raindrops falling to form pits of varying sizes, because with the increase in the PAM-CMC content, the gelation force between soil particles is strengthened to further maintain the integrity of the soil, so the impact of rainfall can only occur on the surface of the soil, and the loss of soil with the runoff is reduced [11,18,53]. In this study, the specimen surface of 2% PAM-CMC showed deeper pits due to raindrops, which were still eroded by the rainwater into the soil interior under strong rainfall. The 3% PAM-CMC specimens still showed craters of varying sizes but did not erode into the soil due to the raindrops, which may be mitigated by the presence of vegetation, whose foliage reduces rainfall scouring and whose root system reduces rainfall erosion [54,55]. The 4% PAM-CMC sample retained its original structure under rainfall, with only a few small craters on the surface due to increased bonding between soil particles. Therefore, for the performance of PAM-CMC in soil erosion resistance studies, 4% is recommended as the optimum application rate.

### 4.5. Effects of PAM-CMC on Plant Growth and Plant Height

In this study, the factors that affected the height of plant growth in terms of their growth conditions were water and the living environment within the soil. As shown in Figure 11, at the early stage of plant growth, plants in the unamended soil germinated and grew rapidly with sufficient water due to the loose soil texture; on the contrary, plants in amended soil grew slowly, probably due to the fact that the soil became less porous and more structurally intact after the addition of PAM-CMC, which resulted in the difficulty of root access to the pore space [56] when the seeds germinated. After 30 days of plant growth, it was clear that the plants in the unamended soil had begun to wilt and yellow their leaves, whereas the plants in the amended soil continued to grow without wilting, suggesting that the PAM-CMC may have reduced the evaporation of water from the soil, which was confirmed by the water absorption and attachment properties of the CMC [9,57]. As can be seen in Figure 12, PAM-CMC may be negative for plant growth in the early stages, but as time increases, plant height begins to exceed the level of natural growth, and in general, the addition of PAM-CMC still promotes plant growth. Previous studies have shown that the addition of appropriate amounts of PAM improves soil structure and reduces runoff while maintaining total nitrogen, available phosphorus, and available potassium levels within a certain range, providing adequate nutrients for vegetation without the need for additional fertilizers [58,59]. However, no improvement was seen in the 4% PAM-CMC specimen. In previous studies, high contents of CMC led to smaller and hardened pores in the soil, a dense soil interior, and reduced air permeability [9]. Therefore, 3% PAM-CMC is recommended as the optimum application rate to ensure that the vegetation can continue to grow in the later stages.

### 4.6. Selection of Optimal PAM-CMC Content and Field Application

In this study, a method for the ecological restoration of high-steep rocky slopes in seasonally frozen areas was proposed. The combined results showed that 3% PAM-CMC was the optimal application rate, which not only significantly increased the cohesion and internal friction angle of the soil, but also improved the soil’s water stability and freeze–thaw and erosion resistance and provided a suitable living space for the plant roots to prolong the plants’ lifetime. When the content of PAM-CMC reached 4%, although the shear strength and erosion resistance of the soil could be further improved, such a high concentration made the soil harden and reduced the pore space, which, in turn, affected the survival of plants [9].

**Figure 14:**
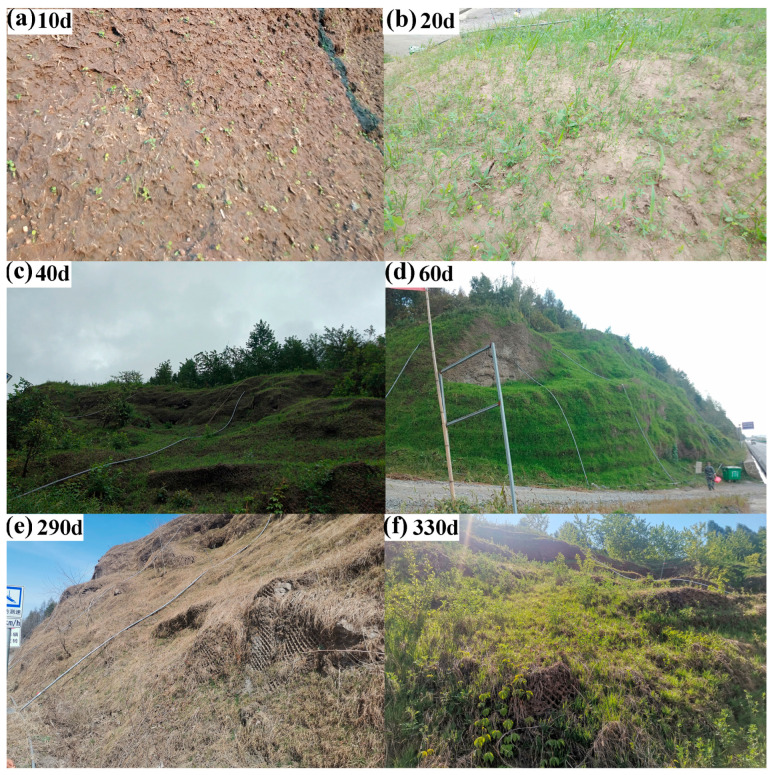
Vegetation growth after spray seeding.

In this study, a 3% PAM-CMC content was applied in the ecological restoration of the study area, and the effectiveness of the optimal PAM-CMC content in practical engineering applications was investigated. Figure 14 reflects the growth of plants after the application of the optimal PAM-CMC content on rocky slopes in the field. The substrate of the slopes was not stripped and lost with the runoff under rainfall due to the additional reinforcement of the wire mesh and anchors in the early stage of spray seeding [9]; the vegetation started to germinate after 10 days of growth (Figure 14a); the average plant height after 20 days of growth (Figure 14b) was 10 cm, with a 40% cover rate; the substrate was stably attached to the high-steep rocky slopes, which was due to the fact that the vegetation roots started to play a reinforcing role [60]; and the system formed by the vegetation–soil further improved the shear strength of the substrate [61,62]. After 40 days of vegetation growth (Figure 14c) with an average plant height of 20 cm and 60% coverage, the vegetation root system continued to develop, in which shrub species began to gradually emerge, which can effectively prevent landslides [63]. After 60 days of vegetation growth (Figure 14d), the average plant height reached 50 cm, the coverage rate was more than 80%, and the well-developed root system of the shrub species was stabilized on the slope surface, which greatly reduced soil erosion [64], and the soil’s water retention and moisture-holding performance was improved, with less artificial rehydration in the later stage. In areas of seasonally frozen soil, where frost heave and thaw settlement are more frequent, the physicochemical properties and survival of vegetation are significantly affected [65], especially in high-steep slopes, where the mechanical reinforcement of soil by vegetation is particularly important [66]. In this study, the soil with 3% PAM-CMC was still able to stably attach to the slope (Figure 14e) after a freeze–thaw cycle of up to 5 months (November–March), with a water content within 16–20%, while the vegetative root system also provided some reinforcement. After the freeze–thaw cycle was over, the vegetation on the slope was able to grow naturally as the temperature rose, and no manual maintenance was required at a later stage (Figure 14f).

After comprehensively analyzing the results of the field application, the PAM-CMC used in this study showed excellent results in terms of soil resistance to freeze–thaw cycles, and the substrate remained stably attached to high-steep rocky slopes after freeze–thaw action, demonstrating the effectiveness and feasibility of the application of PAM-CMC to high-steep rocky slopes in seasonally frozen soil areas. In future research, in areas related to seasonally frozen soils, the mechanism underlying its soil freeze–thaw resistance can be studied to ensure that the content of PAM-CMC can be used more accurately, which can improve the freeze–thaw resistance and, at the same time, make the cost of application more clear.

## 5. Conclusions

In this paper, the shear resistance, water stability, freeze–thaw resistance, and erosion resistance of PAM-CMC in powdery clay soils on high-steep rocky slopes in seasonally frozen areas were investigated through a series of laboratory tests, and the effects of different PAM-CMC contents on plant growth were studied. Based on the results of this study and the discussion and analysis, the following conclusions were drawn about PAM-CMC:

(1) The addition of PAM-CMC was conducive to the improvement of soil shear resistance. When the content of PAM-CMC was ≤3%, it significantly improved the cohesion and internal friction angle of the soil, and when the content of PAM-CMC was 4%, the cohesion of the soil could still be significantly improved, while the improvement of the internal friction angle was not obvious, so, in terms of the soil’s resistance to shear, the optimal amount of PAM-CMC application was 4%.
(2) The water stability, freeze–thaw resistance, and erosion resistance of the soil were significantly improved with an increasing PAM-CMC concentration. The frost heave rate, thaw settlement coefficient, and erosion rate of the soil decreased exponentially with the PAM-CMC content. It is important to note that the water stability of the soil could only be further improved with an appropriate curing time (≥3 days). In the results of the study on the water stability, freeze–thaw resistance, and erosion resistance of the soil, the optimum application rates of PAM-CMC were 3%, 3%, and 4%, respectively.
(3) PAM-CMC affected the preliminary seed germination, which, in turn, slowed down the growth of the plants, but the addition of PAM-CMC prolonged the survival longevity of the plants, which, in combination with previous studies, can be explained by the fact that PAM-CMC improves the soil’s water retention and moisture retention capacity; however, too much PAM-CMC is capable of affecting the height of plant growth. Therefore, the optimum application rate of PAM-CMC is 3% to ensure the late growth of vegetation.
(4) By comprehensively analyzing the results of this study, the optimum application rate of PAM-CMC in this study was determined to be 3%, which not only improved the shear strength, water stability, freeze–thaw resistance, and erosion resistance of the soil but also facilitated the long-term growth of vegetation. Therefore, for field applications, the 3% PAM-CMC soil remains optimal.

## Figures and Tables

**Table 1:** Basic properties of soil samples.

Liquid Limit (%)	Plastic Limit (%)	Optimum Water Content (%)	Maximum Dry Density (g/cm^3^)	Specific Gravity	Natural Water Content (%)
30.9	22.2	18.9	1.6	1.8	18.1

**Table 2:** Properties of CMC and PAM.

	Purity (%)	Density (g/cm^3^)	pH	Viscosity (mPa·s)	W_h_ (%)
CMC	>95	0.591	6.5	52.7	11.5
PAM	>95	0.989	7	15.7	8.1

## Data Availability

The data presented in this study are available upon request from the corresponding author.
